# Supplementary material for: Adaptive Responses of a Peroxidase-like Polyoxometalate-Based Tri-Assembly to Bacterial Microenvironment (BME) Significantly Improved the Anti-Bacterial Effects
Source: Int J Mol Sci. 2023 May 16;24(10):8858. doi: 10.3390/ijms24108858 (PMC10219110; doi:10.3390/ijms24108858)
Supplement: Supplementary file 1 [file ijms-24-08858-s001.zip › ijms-2342546-supplementary.pdf]

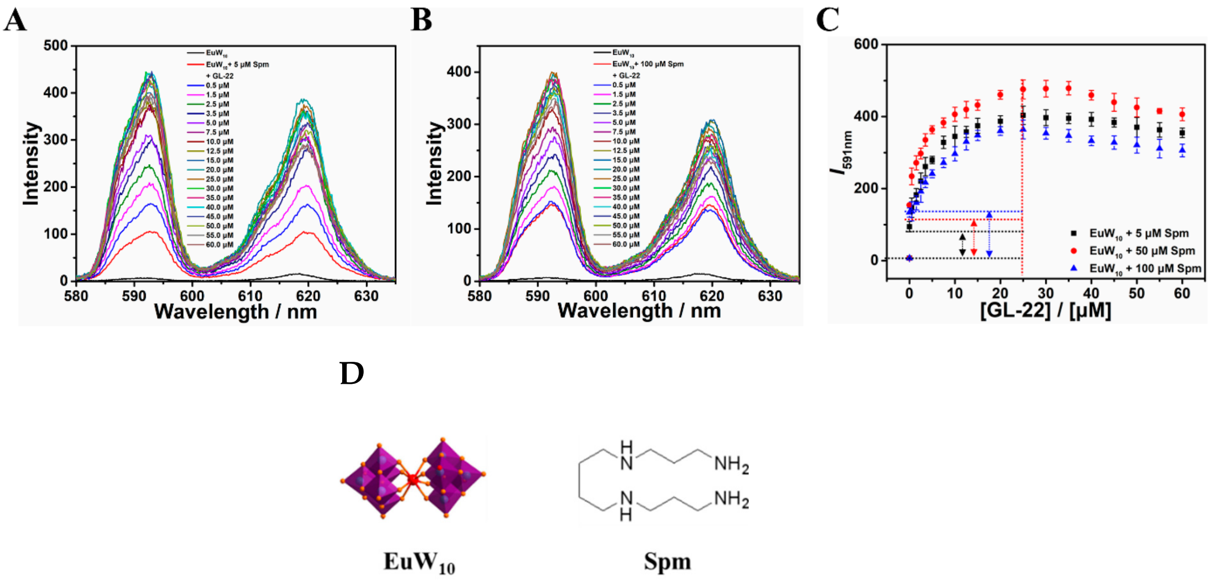

Figure S1. Fluorescence spectra of (A) EuW10/Spm (50 μM/5 μM), and (B) EuW10/Spm (50 μM/100 μM) assembly upon adding different amounts of GL-22 (0–60.0 μM). (C) The plots of the fluorescence intensity changes of EuW10 at 591 nm upon adding different amounts of GL-22 and Spm, respectively. (D) The scheme representation of the EuW10 and spermine.

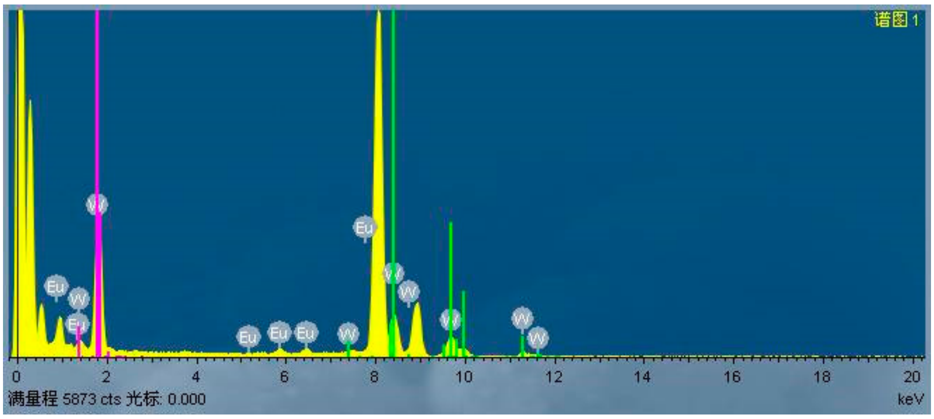

Figure S2. Energy dispersive spectrum of EuW10/Spm/GL-22 (50 μM/50 μM/35 μM) corresponding to Figure 3D.

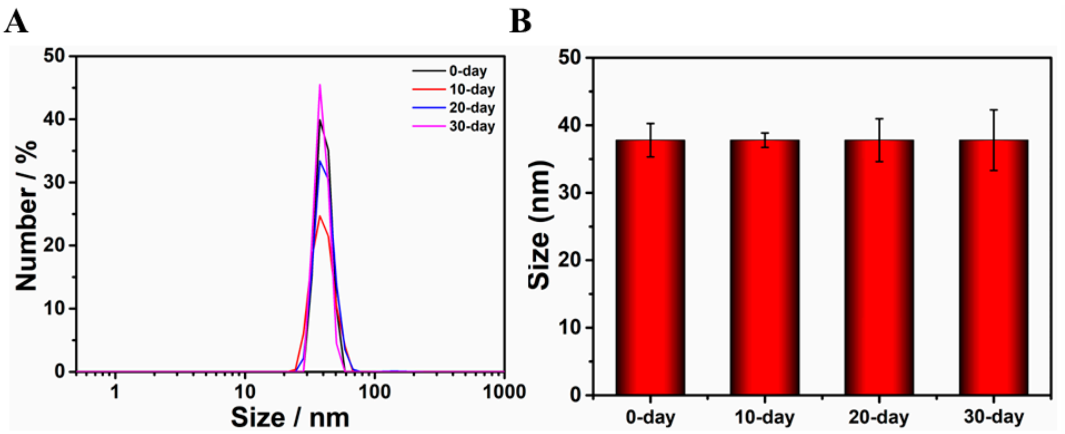

Figure S3. Time-dependent (A) size distributions by DLS and (B) the corresponding histogram of EuW_{10}/Spm (50 μM/50 μM) in buffer solution for 30 days.

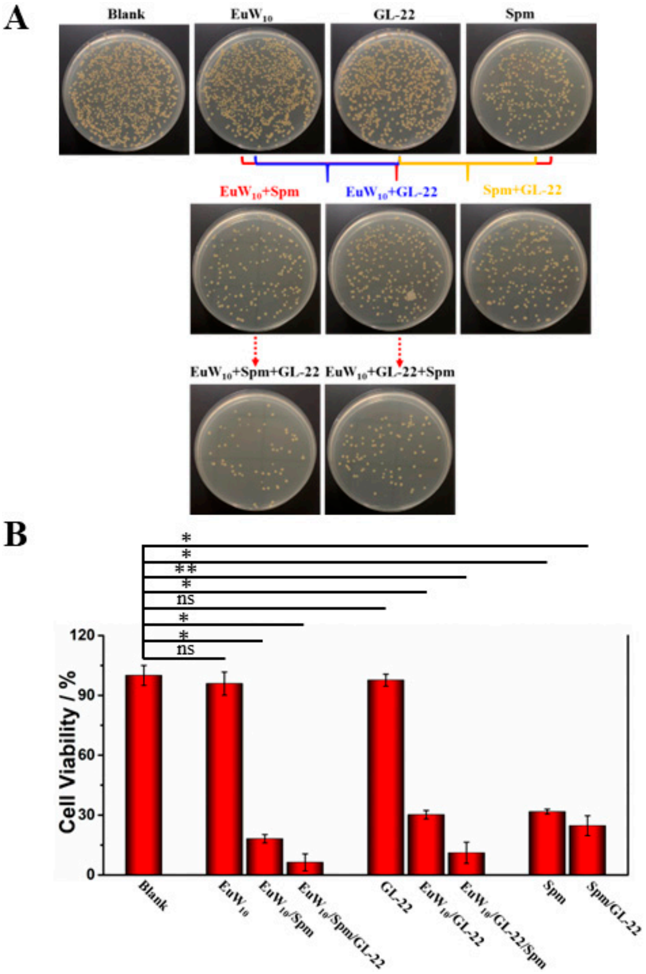

Figure S4. (A) Photographs and (B) statistic viability of S. aureus colonies cultured at 37 °C for 18 h after coating the plates (n=3). Before coating, they were treated with (I) PBS as blank

(Column 1); from up to down of (II) EuW_{10}, EuW_{10}/Spm, and EuW_{10}/Spm/GL-22 (Column 2); (III) GL-22, EuW_{10}/GL-22, and EuW_{10}/GL-22/Spm(Column 3); (IV) Spm, and Spm/GL-22 (Column 4), for 3 h in culture medium, respectively. In all cases, the concentration of EuW_{10}, Spm, and GL-22 is the same as 2.5 μM, 2.5 μM, and 1.25 μM, respectively. Statistical significance was examined using one-way ANOVA analysis: $*p < 0.05$; $**p < 0.01$; "ns" no significance.

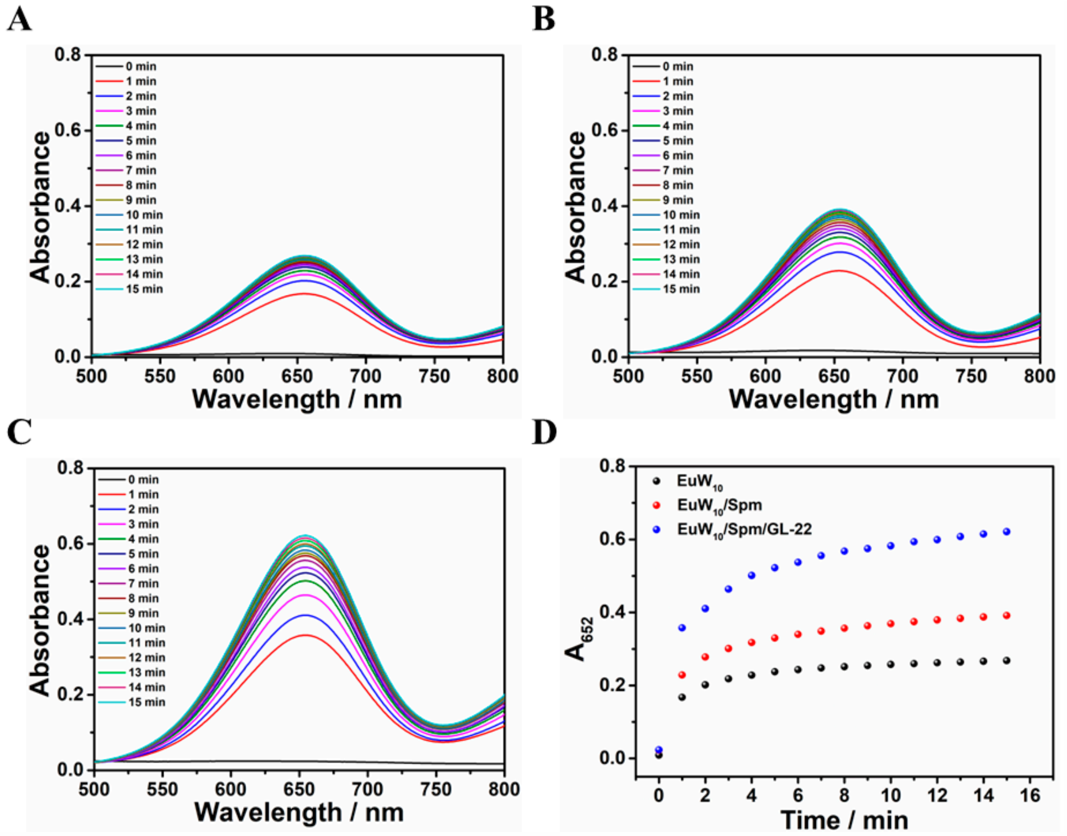

Figure S5. Time-dependent UV-vis absorption spectra of TMB (0.5 mM) in the presence of (A) EuW_{10} (50 μM); (B) EuW_{10}/Spm (50 μM/50 μM); (C) EuW_{10}/Spm/GL-22 (50 μM/50 μM/35 μM). (D) The plots of corresponding intensity changes at 652 nm for (A), (B) and (C).

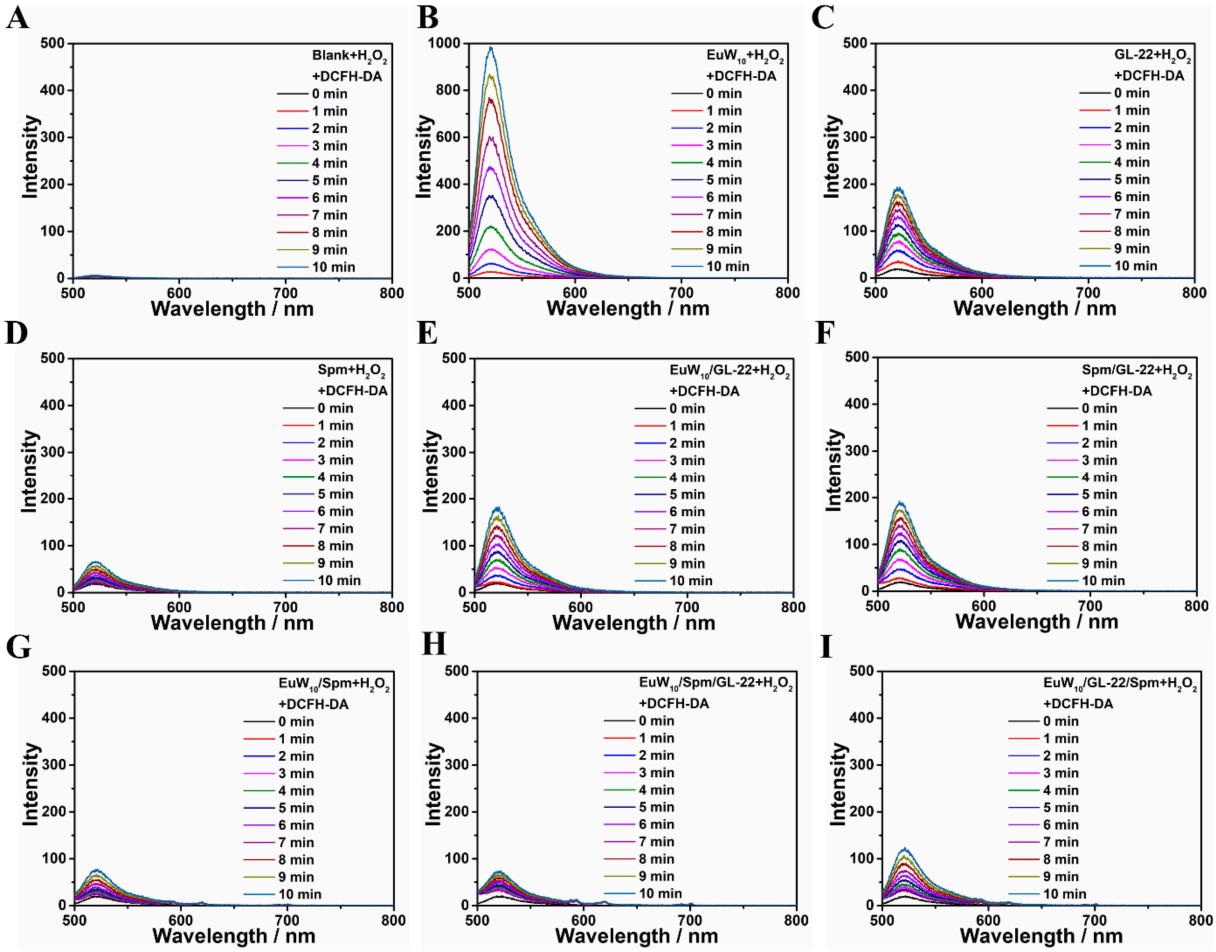

Figure S6. Fluorescence spectra of DCFH-DA (30 μM) in the presence of 10.0 mM H_2O_2 for (A) blank, and those of PBS/culture medium after the treatment with (B) EuW_{10}, (C) GL-22, (D) Spm, (E) EuW_{10}/GL-22, (F) Spm/GL-22, (G) EuW_{10}/Spm, (H) EuW_{10}/Spm/GL-22, (I) EuW_{10}/GL-22/Spm, (λ_{ex} = 488 nm). In all cases, the concentration of EuW_{10}, Spm, and GL-22 was the same as 50 μM, 50 μM, and 25 μM, respectively.

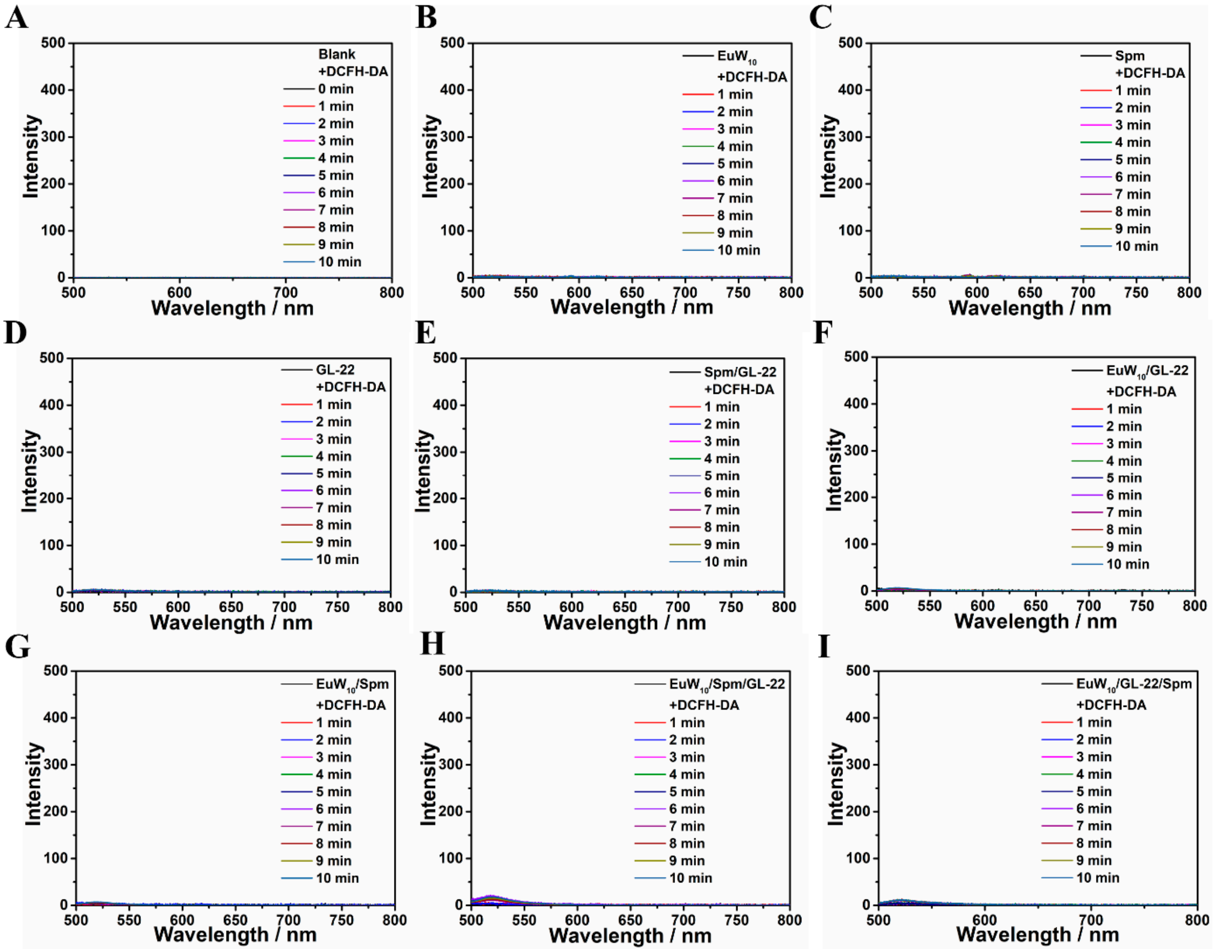

Figure S7. Fluorescence spectra of DCFH-DA (30 μM) in (A) PBS as control, and those in the presence of (B) EuW_{10}, (C) Spm, (D) GL-22, (E) Spm/GL-22, (F) EuW_{10}/GL-22, (G) EuW_{10}/Spm, (H) EuW_{10}/Spm/GL-22, (I) EuW_{10}/GL-22/Spm in PBS, respectively (λ_{ex} = 488 nm). In all cases, the concentration of EuW_{10}, Spm, and GL-22 was the same as 50 μM, 50 μM, and 25 μM, respectively.

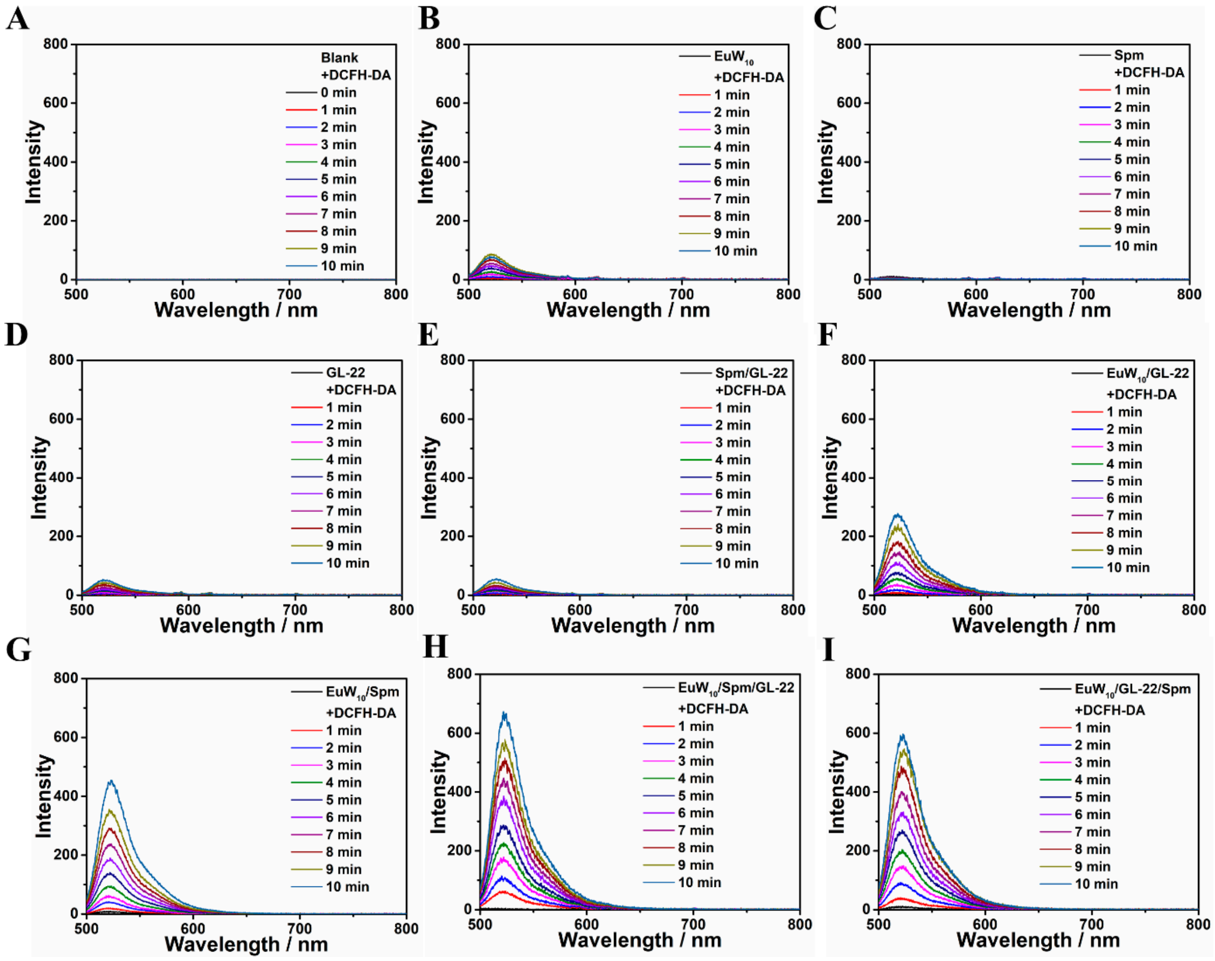

Figure S8. Fluorescence spectra of DCFH-DA (30 μM) in the presence of (A) E. coli as control, and those of E. coli after the treatment with (B) EuW_{10}, (C) Spm, (D) GL-22, (E) Spm/GL-22, (F) EuW_{10}/GL-22, (G) EuW_{10}/Spm, (H) EuW_{10}/Spm/GL-22, (I) EuW_{10}/GL-22/Spm in culture medium, respectively (λ_{ex} = 488 nm). In all cases, the concentration of EuW_{10}, Spm, and GL-22 was the same as 50 μM, 50 μM, and 25 μM, respectively.

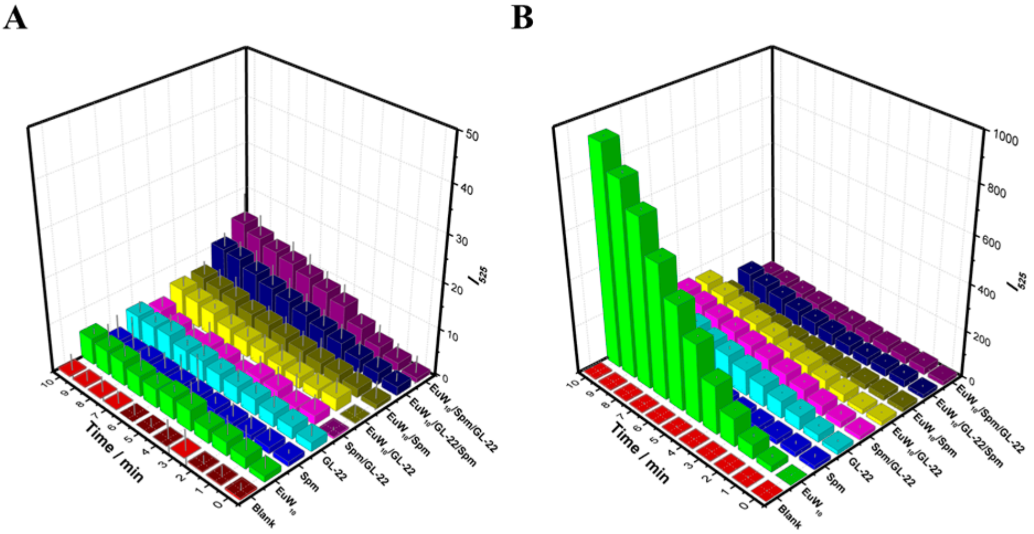

Figure S9. Fluorescence intensity of DCFH-DA (30 μM) at 525 nm for blank, and those of culture medium in the (A) absence and (B) presence of 10.0 mM H_2O_2, after the treatment with EuW_{10}, Spm, GL-22, Spm/GL-22, EuW_{10}/GL-22, EuW_{10}/Spm, EuW_{10}/GL/22-Spm, EuW_{10}/Spm/GL-22, respectively (λ_{ex} = 488 nm). In all cases, the concentration of EuW_{10}, Spm, and GL-22 was the same as 50 μM, 50 μM, and 25 μM, respectively.

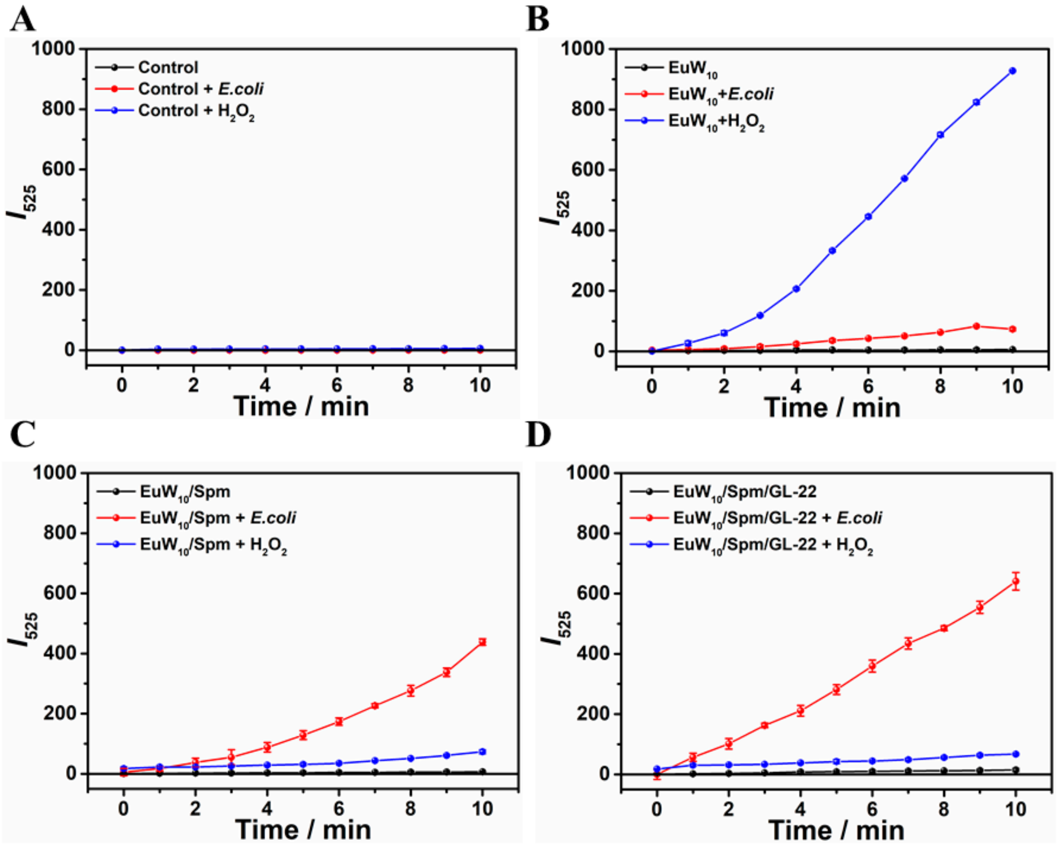

Figure S10. Fluorescence intensity of DCFH-DA (30 μM) at 525 nm for (A) controls, (B) EuW_{10}, (C) EuW_{10}/Spm, and (D) EuW_{10}/Spm/GL-22 before (blank) and after the treatments of E.coli (red) and 10.0 mM H_2O_2 (blue), respectively (λex = 488 nm). In all cases, the concentration of EuW_{10}, Spm, and GL-22 was the same as 50 μM, 50 μM, and 25 μM, respectively.

Table S1. Comparison of MIC results of EuW_{10} assemblies with the reported materials.

Bacteria	Materials	MIC	Time	Ref.
E. coli	Date Syrup Polyphenols	30(±0.83) mg·mL^{-1}	16 h	[30]
S. aureus	Date Syrup Polyphenols	30(±076) mg·mL^{-1}	16 h	[30]

E. coli	antimicrobial peptide P-113	>64 μg·mL^{-1}	16 h	[31]
S. aureus	antimicrobial peptide P-113	32 μg·mL^{-1}	16 h	[31]
S. aureus	vancomycin	1 mg·L^{-1}	16 h	[32]
E. coli	Cefquinome	0.25 μg·mL^{-1}	72 h	[33]
S. aureus	Cefquinome	1μg·mL^{-1}	72 h	[33]
S. aureus	PAMAMG5 NPs	8 μg·mL^{-1}	24 h	[34]
E. coli	PAMAMG5 NPs	4 μg·mL^{-1}	24 h	[34]
E. coli	bamboo charcoal/polyoxometalate	0.2–2 mg·mL^{-1}	18 h	[35]
S. aureus	bamboo charcoal/polyoxometalate	0.02–2 mg·mL^{-1}	18 h	[35]
E. coli	EuW_{10}	167.55 μg·mL^{-1}	16 h	This work
S. aureus	EuW_{10}	251.38 μg·mL^{-1}	16 h	This work
E. coli	EuW_{10}/Spm/GL-22	41.88 μg·mL^{-1}	16 h	This work
S. aureus	EuW_{10}/Spm/GL-22	20.94 μg·mL^{-1}	16 h	This work

Table S2. The viabilities of E-coli colonies after being treated with different assemblies, for 1–9 h.

	1 h	3 h	9 h		1 h	3 h	9 h
Control	100	100	100.05	Control	100	100	100.05
EuW_{10}	99.73	90.26	57.16	EuW_{10}	99.73	90.26	57.16
EuW_{10}/Spm	96.06	83.09	39.43	EuW_{10}/GL-22	91.67	80.88	35.14
+ 5 μM GL-22	90.40	79.41	32.57	+ 5 μM Spm	85.52	78.09	25.28
+ 15 μM GL-22	77.67	54.21	21.65	+ 25 μM Spm	61.94	33.16	0.5179
+ 25 μM GL-22	68.07	48.58	13.26	+ 50 μM Spm	65.43	45.78	5.771
+ 35 μM GL-22	61.61	40.73	0.5090	+ 75 μM Spm	70.85	51.47	10.19
+ 50 μM GL-22	63.36	58.42	3.194	+ 100 μM Spm	71.64	53.74	11.40
+ 70 μM GL-22	74.55	73.93	8.952				
